# Previously defined 53 immune gene panel predicts melanoma survival using The Cancer Genome Atlas (TCGA)

**DOI:** 10.1186/2051-1426-3-S2-P283

**Published:** 2015-11-04

**Authors:** Robyn Gartrell, Qian Yingzhi, Lopez Gonzalo, Fu Yichun, White-Stern Ashley, Bansal Mukesh, Sivendran Shanthi, Califano Andrea, Chang Rui, Saenger Yvonne

**Affiliations:** 1 Columbia University/New York Presbyterian, New York, NY, USA; 2 Columbia University, New York, NY, USA; 3 Department of Systems Biology at Columbia University, New York, NY, USA; 4 Columbia University College of Physicians of Surgeons, New York, NY, USA; 5 Lancaster General Health, Hummelstown, PA, USA; 6 Icahn School of Medicine at Mount Sinai Hospital, New York, NY, USA

## Background

Our laboratory has discovered a 53-immune gene panel predictive of survival in patients with stage II-III melanoma[1]. Validation is planned using tissues from a multi-institutional cooperative group study. We tested the 53-immune gene panel using publicly available data for melanoma from The Cancer Genome Atlas (TCGA).

## Methods

TCGA dataset for melanoma (n=407) was screened to determine two extreme groups of patients – alive at least 4 years (n=67) and dead within 2 years from diagnosis (n=59). Genome wide differential expression analysis was performed between the two groups to study our prognostic signature. We used gene set enrichment analysis (GSEA) to evaluate the activation status of the 53 gene panel as well as multiple immune lineage specific gene sets[2]. We used PAM clustering algorithm to define two groups based on the expression data of the 53 genes and performed survival analysis on the unsupervised patient clusters.

## Results

**Figure 1:**
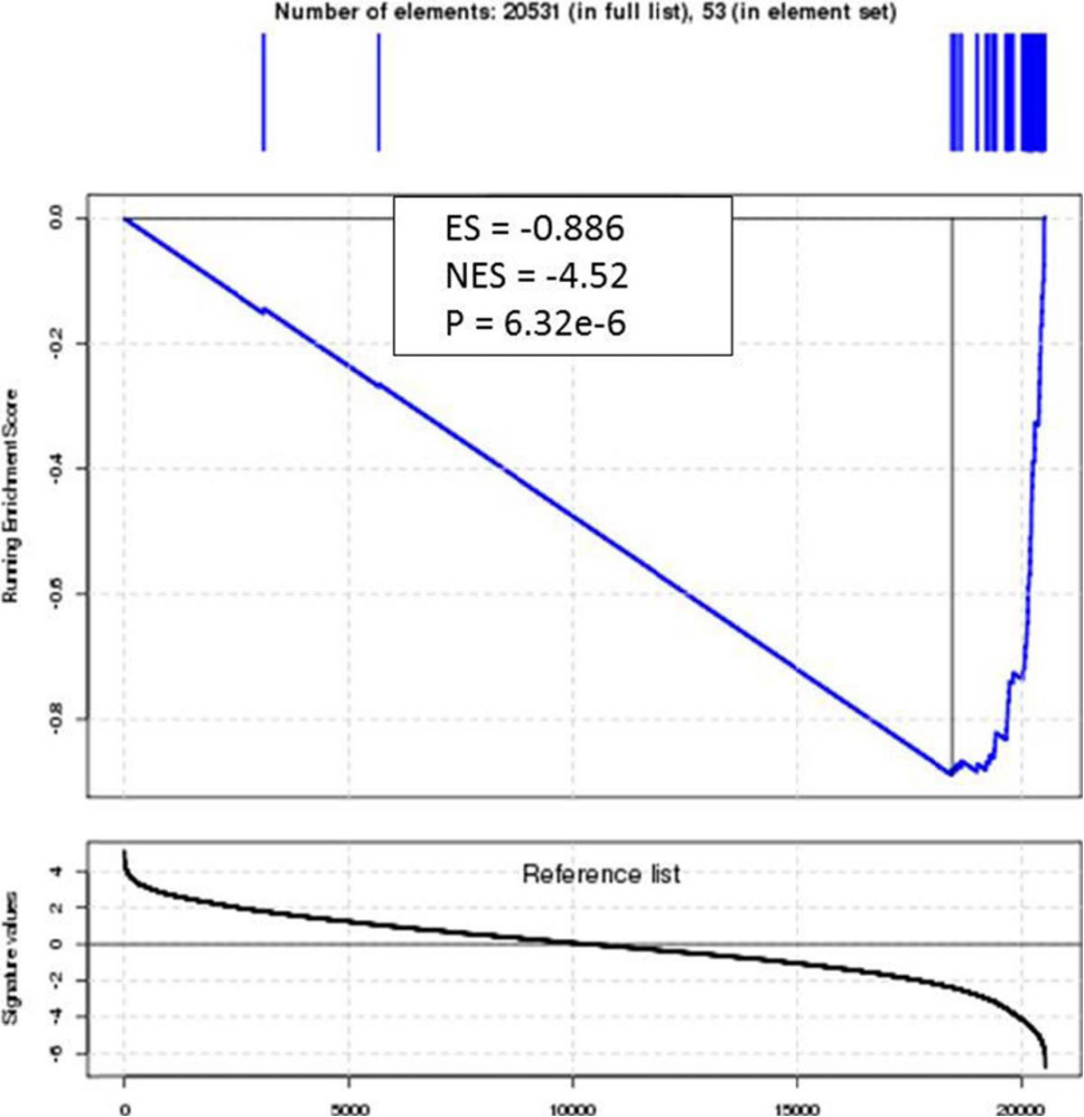


**Figure 2:**
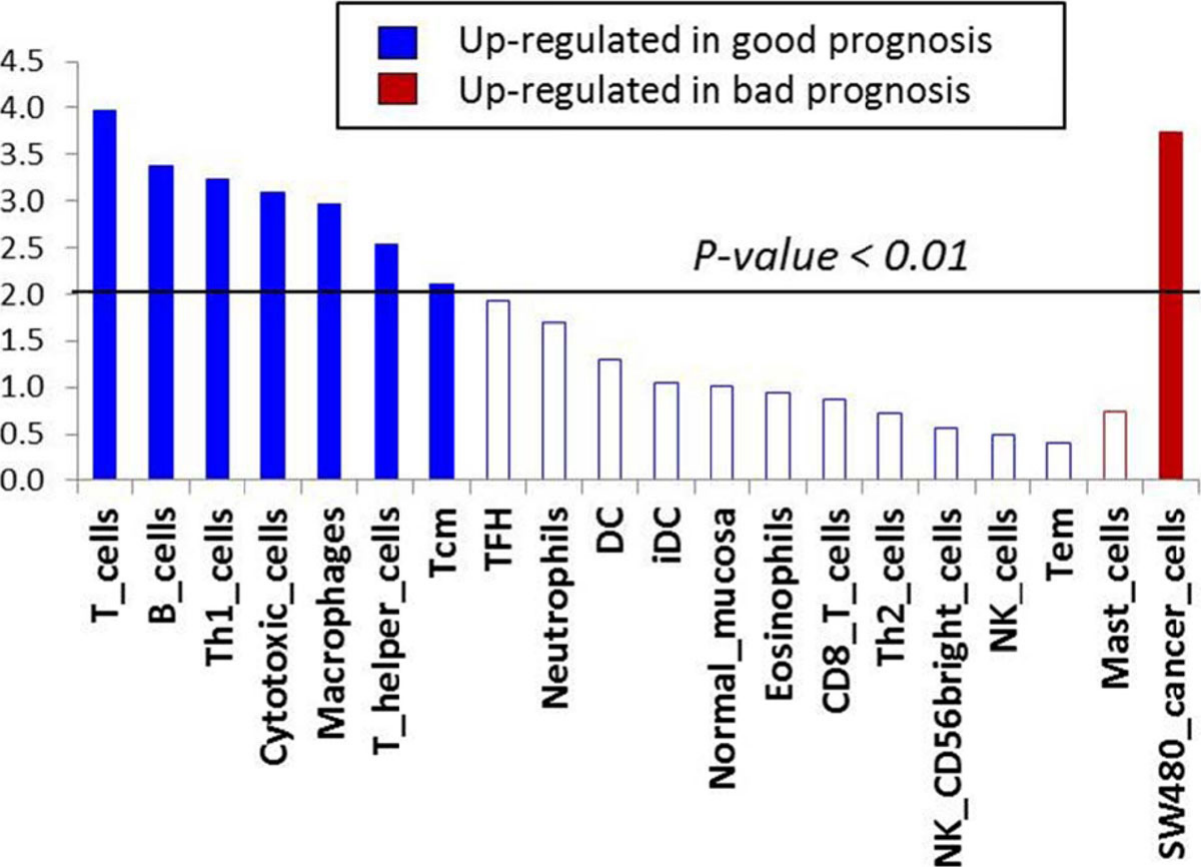


**Figure 3:**
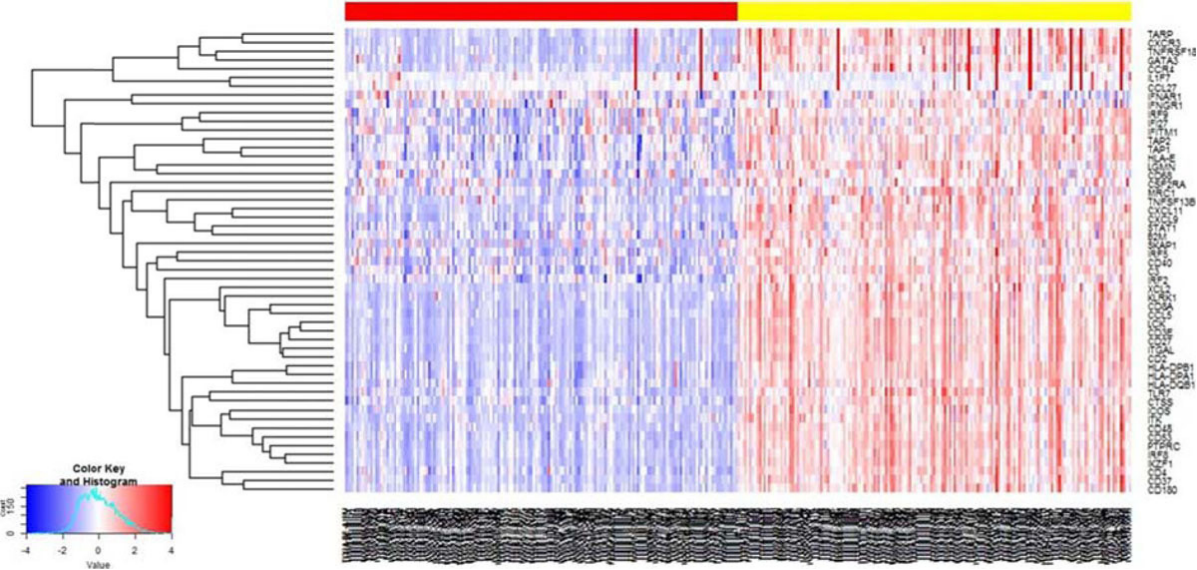


Prognostic relevance of the 53 gene panel was tested using supervised and unsupervised approaches. First, the prognostic signature shows striking GSEA results (p=2.24e-5) with activation of 51 of the 53 immune genes in good prognosis patients, indicated by a right shift in the enrichment score (Figure 1). GSEA of immune lineage specific gene sets^2^ is shown in Figure 2. Using an unsupervised approach we studied the 53 gene panel across the whole population of melanoma cases with clinical and expression data reported in the TCGA (n=336). The generated heat map shows two distinct clusters of expression (Figure 3). A Kaplan Meier curve shows overall survival at 10 years in Cluster 1: 31.4 % and Cluster 2: 45.6%, (p =0.00261).

## Conclusions

Using publically available data from TCGA we tested the 53-immune gene panel using supervised and unsupervised approaches, showing that 51 of the 53 genes are predictive of survival. This shows that the immune genes may be operative in different stages of melanoma. We propose using this panel to evaluate the effect of immunotherapy across melanoma cohorts, and have begun this analysis. We will also be evaluating the use of the 53 immune genes and the specific immune gene subsets on other cancer populations.
